# Association of Lipid and Body Mass Index Profile With Chronic Hepatitis C Infection Stratified by Age and Gender

**DOI:** 10.7759/cureus.20665

**Published:** 2021-12-24

**Authors:** H Maqsood, Tamoor Chughtai, Abdul Basit Khan, Shifa Younus, Akifa Abbas, Usman A Akbar, Shaheryar Qazi

**Affiliations:** 1 Internal Medicine, Nishtar Medical University, Multan, PAK; 2 Medicine, Nishtar Medical University, Multan, PAK; 3 Infectious Diseases, University of Louisville, Louisville, USA

**Keywords:** hepatitis c, lipid profile, bmi, dyslipidemia

## Abstract

Background

In this study, we aimed to determine the association of lipid and body mass index (BMI) profiles among cases having chronic hepatitis C virus (CHCV) infection.

Methodology

This cross-sectional study was conducted in the outpatient department of a tertiary care hospital. A total of 320 cases of both genders, aged 18 to 60 years, with CHCV infection were enrolled in the study. After obtaining relevant history and conducting a physical examination, the venous blood sample of each patient was taken and sent to the institutional laboratory to analyze serum total cholesterol, serum triglyceride, low-density lipoprotein, and high-density lipoprotein levels. BMI of all the study participants was also noted.

Results

Of the total 320 cases, there were 152 (47.5%) males and 168 (52.5%) females. The overall mean age was 42.92 ± 11.38 years. Most cases [97 (30.3%)] were in the 41 to 50-year age group. Overall, the mean BMI was 27.75 ± 4.59 kg/m^2^. Dyslipidemia was noted in 144 (45.0%) cases. Increasing age and increasing BMI were found to have statistical significance with the presence of dyslipidemia (p < 0.05).

Conclusions

Increasing age and BMI have a significant association with dyslipidemia in patients with CHCV infection. Lipid profile appears to differ among different age and BMI groups.

## Introduction

Hepatitis, particularly among developing countries, is a prime global health concern. Hepatitis is an inflammation of the liver due to infection with hepatitis viruses, mainly the B (HBV) and C (HCV) types [1]. It is estimated that HCV infects approximately 185 million people worldwide, making it a crucial cause of morbidity and mortality [2]. Every year more than one million patients with hepatitis infection die because of complications such as cirrhosis and hepatic carcinoma [3]. Globally, in the general population, the prevalence of HCV varies from 1.3% to 2.9%, while it is estimated to be approximately 6.5% in Pakistan [4,5].

After testing positive for the disease for more than six months, infection with chronic hepatitis C virus (CHCV) is defined as a non-remission disease. In the acute phase of the disease, 70-90% of HCV-infected cases do not achieve spontaneous clearance of the virus and become chronically infected with HCV [6]. CHCV infection is a major contributor to liver cirrhosis and its complications such as hepatic encephalopathy, variceal bleeding, and ascites [7]. It has been estimated that approximately 27% of liver cirrhosis cases and 25% of liver cancer cases worldwide are due to CHCV infection [8].

The liver is the main organ where lipoprotein formation and clearance occurs. The liver receives fatty acids and cholesterol from the peripheral tissues and diet, and then modules them as lipoprotein complexes and releases them into circulation. Hence, all the major liver diseases affect lipid metabolism [9,10].

A large-scale study conducted in the United States indicated that among individuals with CHCV infection, 70.5% were afflicted with hyperlipidemia [11]. Some studies have suggested that plasma cholesterol levels reduce in CHCV infection [12]. The body mass index (BMI) has been shown to have a strong correlation with the fibrosis index and cirrhosis at the time of presentation among patients with CHCV infection [13]. In addition, previous studies have reported that clinical manifestations of metabolic syndrome influence the progression of CHCV infection [14].

Limited literature exists regarding the association of lipid profile and BMI with chronic hepatitis C virus infection while a few local studies have been conducted. Our study sought to evaluate the association of lipid and BMI profiles in patients with CHCV infection. The findings of this study will aid in the monitoring and potential management of lipid profile and BMI when cases of CHCV infection are encountered in daily healthcare practice.

## Materials and methods

Study setting

This study was conducted in the outpatient department of a tertiary care hospital in Multan from August 2020 to January 2021.

Study participants, sample size, and sampling technique

A total of 320 patients of both genders with CHCV infection were approached for inclusion in the study. Simple random sampling was performed.

Study design

The research approach employed a cross-sectional, observational design to evaluate the association of lipid and BMI profiles with CHCV infection in relation to age and gender.

Inclusion and exclusion criteria

Patients with CHCV infection with continuously detectable hepatitis C RNA on polymerase chain reaction tests for at least one year were included in the study. Patients with acute hepatic disease, kidney disease, ischemic heart disease, or any other acute or chronic disorder were excluded. In addition, patients taking lipid-lowering drugs, systemic or inhaled glucocorticoids, or any other medications known to interfere with lipid metabolism were also excluded.

Data collection procedure

After obtaining consent from the ethical committee of the hospital, we conducted this study in the outpatient department of the hospital. We explained the objectives of the study to the participants and took their consent. After obtaining relevant history and conducting a physical examination, the venous blood sample of each patient was drawn and sent to the institutional laboratory to analyze serum total cholesterol (TC), serum triglyceride (TG), low-density lipoprotein (LDL), and high-density lipoprotein (HDL) levels. Dyslipidemia was considered if any one of the following was noted: TC >200 mg/dL, TG >150 mg/dL, HDL <40 mg/dL in males and <50 mg/dL in females, or LDL >130 mg/dL. BMI of all the study participants was also noted. We designed a specialized proforma to manage all the study information.

Data analysis

All data were analyzed using SPSS version 25.0 (IBM Corp., Armonk, NY, USA). The data were reported as means ± standard error. Frequencies and percentages were noted for gender and frequency of dyslipidemia. The mean and standard deviation were estimated for age, BMI, duration of CHCV infection, and TC, TG, LDL, and HDL levels. We used the Student’s t-test to compare the quantitative variables, while the chi-square test was used to compare qualitative variables. A p-value of <0.05 was considered statistically significant.

## Results

Of the total 320 cases, there were 152 (47.5%) males and 168 (52.5%) females. The age range was 18 to 60 years, and the overall mean age was 42.92 ± 11.38 years. Most cases [97 (30.3%)] were in the 41 to 50-year age group. Overall, the mean BMI was 27.75 ± 4.59 kg/m^2^, while most cases [167 (52.2%)] had a BMI between 25 and 30 kg/m^2^. Dyslipidemia was noted in 144 (45.0%) cases. Table 1 shows the demographics and clinical characteristics of the study participants.

**Table 1:** Demographic and clinical characteristics of the study participants (n = 320).

	n (%)
Gender	
Male	152 (47.5%)
Female	168 (52.5%)
Age range (years)	
18–30	58 (18.1%)
31–40	79 (24.7%)
41–50	97 (30.3%)
51–60	86 (26.9%)
Body mass index (kg/m^2^)	
<25	65 (20.3%)
25–30	167 (52.2%)
>30	88 (27.5%)
Dyslipidemia	
Yes	144 (45.0%)
No	176 (55.0%)

Table 2 shows the stratification of dyslipidemia concerning study characteristics. Age and BMI were noted to have statistical significance with dyslipidemia as increasing age (p = 0.005) and BMI (p < 0.001) were found to have statistical significance with the presence of dyslipidemia (p < 0.05).

**Table 2:** Stratification of dyslipidemia with respect to the study variables.

		Dyslipidemia		P-value
		Present (n = 144)	Absent (n = 176)	
Gender	Male	66 (45.8%)	86 (48.8%)	0.5891
	Female	78 (54.2%)	90 (51.2%)	
Age (years)	18–30	18 (12.5%)	40 (22.7%)	0.005
	31–40	28 (19.4%)	51 (29.0%)	
	41–50	54 (37.5%)	43 (23.9%)	
	51–60	44 (30.6%)	42 (23.9%)	
Body mass index (kg/m^2^)	<25	26 (18.1%)	39 (22.2%)	<0.001
	25–30	60 (41.7%)	107 (60.8%)	
	>30	58 (40.3%)	30 (17.0%)	

TC and TG levels were significantly high in cases between 41 and 60 years of age (p < 0.05). HDL level was significantly low in the 41 to 60-year age group (p = 0.033). Table 3 shows the stratification of the mean lipid profile concerning age and gender.

**Table 3:** Stratification of the mean lipid profile with respect to gender and age. TC: total cholesterol; TG: triglycerides; LDL: low-density lipoprotein; HDL: high-density lipoprotein

Lipid profile	Age		P-value
	18–40 years (n = 137)	41–60 years (n = 183)	
TC (mean ± SD)	141.23 ± 35.10	152.03 ± 38.86	0.011
TG (mean ± SD)	95.24 ± 39.71	106.17 ± 41.29	0.018
LDL (mean ± SD)	100.02 ± 29.24	105.56 ± 35.59	0.138
HDL (mean ± SD)	53.47 ± 11.43	50.99 ± 9.31	0.033
Lipid profile	Gender		
	Male (n = 152)	Female (n = 168)	
TC (mean ± SD)	147.21 ± 37.90	147.58 ± 37.48	0.930
TG (mean ± SD)	97.62 ± 40.98	105.00 ± 40.66	0.107
LDL (mean ± SD)	106.52 ± 32.02	100.18 ± 33.84	0.087
HDL (mean ± SD)	52.17 ± 10.19	51.95 ± 10.47	0.850

Table 4 shows the stratification of BMI with age and gender. No significant difference was noted among study participants between the groups (p > 0.05).

**Table 4:** Stratification of BMI with respect to gender and age. BMI: body mass index

BMI (kg/m^2^)	Age		P-value
	18–40 years (n = 137)	41–60 years (n = 183)	
<25	33 (24.1%)	32 (17.5%)	0.065
25–30	75 (54.7%)	92 (50.3%)	
>30	29 (21.2%)	59 (32.2%)	
BMI (kg/m^2^)	Gender		
	Male (n = 152)	Female (n = 168)	
<25	29 (19.1%)	36 (21.4%)	0.563
25–30	77 (50.7%)	90 (53.6%)	
>30	46 (30.3%)	42 (25.0%)	

## Discussion

The liver is known to be the main organ hosting synthesis, storage, as well as oxidation of lipids and several other types of macromolecules. Lipid metabolism in the liver is vital for maintaining systemic nutrient hemostasis. Disturbance in normal lipid metabolism in the liver is a key characteristic of various diseases such as diabetes mellitus, alcoholic and non-alcoholic fatty liver disease, and viral infections such as HCV infection [15,16]. Chronic liver disease is known to alter the natural lipid metabolism, and some researchers have reported an association between CHCV infection and lipid metabolism [10,12].

We noted age and BMI to have a significant association with dyslipidemia as increasing age and BMI were found to have statistical significance with the presence of dyslipidemia (p < 0.05). Moreover, TC and TG were significantly high in participants aged 41 to 60 years (p < 0.05). However, HDL was significantly low in the 41 to 60-year age group (p < 0.05). A study by Agbecha et al. from Nigeria reported significantly low HDL among cases with CHCV infection [17]. Furthermore, Maggi et al., Serfaty et al., and Fabris et al. noted patients with CHCV infection to have abnormally low LDL levels [18-20]. Floris-Moore et al. noted patients with CHCV infection to have significantly altered cholesterol levels in comparison to controls [21]. The findings of Li et al. were also consistent with our findings. They reported lower HDL levels among cases with CHCV infection [22].

In the present work, we also noted a significant association between dyslipidemia and increasing age. These findings are consistent with the local published data where the authors noted age more than 41 years to be associated with increased prevalence and disturbances in lipid profile [23]. The same study also found HCV infection to be positively associated with increasing BMI, which correlates well with our findings. Kallwitz et al. observed BMI of more than 30 to be associated with histologic progression and cirrhosis among cases with CHCV infection [13].

A few underlying mechanisms have been presented for abnormal lipid metabolism among cases with CHCV infection, but genotypes do not seem to alter these findings. Some researchers have described CHCV infection and its related inflammatory role to contribute to alterations in LDL metabolism, thus affecting atherogenesis [24]. Earlier studies have also shown LDL receptor (LDL-R) having a role in the cellular entry of HCV, while some others have postulated that certain key components of lipoprotein and cholesterol metabolism are involved in the initial entry and infection of HCV [25]. The lifecycle of HCV in the liver also depends upon hepatic cholesterol as well as lipogenesis pathways. Abnormalities in the lipid profile of CHCV patients can also contribute to hepatic steatosis and deposition of hepatocellular lipid droplets [26].

Our study has a few limitations. We could not record the viral load of HCV among our study participants. We were also unable to analyze the fasting samples of our study participants. Alanine transaminase levels were also not noted for the study participants. As we did not note the findings of treatment or management protocols, we are unable to suggest the impact of the management of lipid disorders in patients with CHCV infection on the overall outcome of these patients.

## Conclusions

Increasing age and BMI have a significant association with dyslipidemia in patients with CHCV infection. Lipid profile appears to be altered among different age and BMI groups. Further studies with longer follow-ups and improved study designs will further help us improve our current understanding of CHCV infection with lipid profile and related health issues.
